# ACTN1 interacts with ITGA5 to promote cell proliferation, invasion and epithelial-mesenchymal transformation in head and neck squamous cell carcinoma

**DOI:** 10.22038/IJBMS.2022.67056.14703

**Published:** 2023-02

**Authors:** Rui Wang, Ying Gao, Huimin Zhang

**Affiliations:** 1 Department of Otolaryngology-Head and Neck Surgery, Shanxi Bethune Hospital, Shanxi Academy of Medical Sciences, Tongji Shanxi Hospital, Third Hospital of Shanxi Medical University, Taiyuan, Shanxi, 030032, China; 2 Tongji Hospital, Tongji Medical College, Huazhong University of Science and Technology, Wuhan, 430030, China

**Keywords:** α-Actinin 1, Epithelial-mesenchymal – transformation, Head and neck squamous - cell carcinomas, Invasion, ITGA5

## Abstract

**Objective(s):**

The aim of this study was to detect the expression levels of α-Actinin 1 (ACTN1) and ITGA5 in HNSCC and to explore how ACTN1/ITGA5 regulated the proliferative and invasive abilities, as well as the EMT of Head and neck squamous cell carcinoma (HNSCC) cells.

**Materials and Methods:**

The viability, proliferative, invasive and migrative abilities of HNSCC cells after transfection were, in turn, detected by CCK8 assay, colony formation assay, EdU staining, transwell, as well as wound healing. E-cadherin in transfected cells was assessed utilizing immunofluorescence. RT-qPCR confirmed the transfection effect of ACTN1 and ITGA5 in HNSCC cells and the interaction between ACTN1 and ITGA5 in HNSCC cells was determined by co-immunoprecipitation (Co-IP). With Western blot application, the contents of ACTN1, ITGA5, proliferation-, invasion- and migration-related proteins were estimated. A xenograft model based on nude mice was conducted and Ki-67 content in tumor tissues was evaluated employing immunohistochemistry (IHC) staining.

**Results:**

ACTN1 interacted with ITGA5. The contents of ACTN1 and ITGA5 were found to be abundant in HNSCC tissues and cells and associated with poor prognosis. ACTN1 depletion imparted suppressive impacts on cell proliferative, invasive and migrative abilities as well as EMT of HNSCC cells, which were reversed by ITGA5 overexpression. In addition, ACTN1 deficiency repressed the growth and metastasis of tumor tissues in tumor xenografts of nude mice.

**Conclusion:**

ACTN1 positively interacts with ITGA5 to promote proliferation, invasion and EMT of HNSCC cells. Also, ACTN1 promotes tumor growth and metastasis.

## Introduction

Head and neck squamous cell carcinoma (HNSCC), which accounts for more than 90% of head and neck tumors, ranks as the sixth most frequent malignancy and contributes to more than 890,000 new cases and 450,000 deaths annually in every corner of the world (1, 2). In spite of tremendous advancements in surgery, radiotherapy, and chemotherapy in the past few decades, the 5-year survival rate of patients suffering from HNSCC is discontented; evenly, 70% of patients will exhibit varying degrees of recurrence as well as lymph node metastasis (3, 4). HNSCC is a heterogeneous epithelial tumor closely associated with smoking and alcohol consumption (5). The pathogenesis of HNSCC is complicated, involving the interaction of multiple molecules with cells. Due to the number of sites involved, ideal specific biomarkers are still lacking. Screening HNSCC-related genes are expected to provide new ideas for diagnosis and treatment and offer underlying biomarkers as well as targeted therapies for HNSCC.

α-Actinin 1 (ACTN1) protein belongs to the spectrin gene superfamily, which binds to actin. So far, a total of four α-Actinin subtypes have been identified, which are formed by selective splicing of genes, respectively ACTN1-4. Each subtype plays a specific role in different cell types (6, 7). ACTN1 and ACTN4 are mainly distributed around the microfilaments of non-muscle cells and are attached to the membrane by actin coupling. ACTN2, as well as ACTN3, are predominantly found in muscle cells, except for a few α-Actinin 2 (8). Downregulation of ACTN1 can reduce surgical site implantation to improve the survival rate of mice with colon carcinomas (9). ACTN1 imparts promotive impacts on tumor growth in hepatocellular carcinoma (10). Oroxylin A inhibits ACTN1 expression, inactivates cancer-related fibroblasts, and represses breast cancer metastasis (11). ACTN1 upregulation is related to poor prognosis, and ACTN1 knockdown inhibits cell proliferative ability and metastasis of oral squamous cell carcinoma (OSCC) (12). Nevertheless, the function of ACTN1 in HNSCC needs to be further investigated. 

Results on the LinkedOmics database (http://www.linkedomics.org/login.php) show that ACTN1 is positively correlated with integrin α5 (ITGA5), and STRING (https://cn.string-db.org/) indicates that ACTN1 can interact with ITGA5. ITGA5 is a member of the integrin protein family (13). A previous study has evidenced that high levels of ITGA3, ITGA5 and ITGA6 are associated with poor overall survival in patients with HNSCC, and IGTA5 may act as a pivotal independent prognostic factor (14). ZNF750 can suppress angiogenin, VEGF, RGS5 as well as CD105, inhibit ITGA5, ITGB1 and CD44, and ascend PHD2 as well as PDGFB. ZNF750 upregulation diminishes the viability as well as lateral migrative abilities of three oral squamous cell carcinoma cell lines (15). ITGA5 expression is upregulated in tongue squamous cell carcinoma (TSCC) and the knockdown of ITGA5 suppresses the proliferative, migrative and invasive capabilities of TSCC cells (16). ITGA5 is also elevated in OSCC and ITGA5 promotes the proliferative, migrative and invasive capabilities of OSCC cells (17). Nevertheless, the role that ITGA5 plays in HNSCC has not been clarified.

The present study aimed to detect the expression levels of ACTN1 and ITGA5 in HNSCC and to explore their regulatory effect on cell proliferative and invasive capabilities as well as epithelial-mesenchymal transformation (EMT) of HNSCC cells.

## Materials and Methods

***Cell culture and transfection***

Human immortalized oral epithelial cell (HIOEC) line and Cal27 and SCC9 cell lines were obtained from Procell (Wuhan, China) and Tu686 cell line was brought from BeNa Culture Collection (BNCC, Henan, China). Cancer cells were cultivated in RPMI-1640 medium which was decorated with 10% FBS and 1% antibiotics. Defined Keratinocyte-SFM (Gibco) was utilized for the cultivation of HIOEC cells. The culture condition was 5% CO_2_ at 37°C in a humidity incubator.

The short hairpin RNA (shRNA)-ACTN1, shRNA-NC, and overexpression plasmid vectors targeting ITGA5 (Ov-ITGA5) and Ov-NC were constructed by GenePharma (Shanghai, China). The transfection of the above plasmids into Tu686 cells was implemented by applying Lipofectamine 3000 (Invitrogen, USA) in light of recommended protocol.

***Xenograft model***

Ten male BALB/C nude mice (SPF grade, 18-20 g, 4 weeks old) provided by Beijing Charles River Animal Co, Ltd (China) were separated into two groups (n=5): shRNA-NC group and shRNA-ACTN1 group. A total of 2 × 10^6^ Tu686 cells with or without ACTN1 interference were injected into the back next to the right forelimb of the nude mouse subcutaneously, which were raised with free access to food and water for 21 days. The weights for the rat were measured every 3 days with the electronic balance and tumor volumes were measured every 3 days with the vernier caliper. After that, the tumors in the sacrificed mice were harvested from the surrounding tissues. The weight of tumor was weighted by the electronic balance. All experiments got approval from the Animal Care and Use Committee and the Animal Ethics Committee of Shanxi Bethune Hospital (SBQDL-2022-011).

***RTqPCR***

Total RNA was separated from Tu686 cells and tumor tissues by 1 ml TRIzol® reagent (Shanghai Tronsai Technology Co, Ltd). The purity of the sample was determined by the value of OD260/OD280, and 2 μl of the collected RNA was diluted to 120 μl with pure water without RNA enzyme, and the value of OD260/OD280 was measured by spectrophotometer. When the ratio is between 1.8 and 2.0, it indicates good purity. Total RNA was reverse synthesized into cDNA applying a HiFiScript cDNA Synthesis Kit (Shanghai Yanjin Biotechnology Co, Ltd.) and qPCR was performed using UltraSYBR Mixture (Shanghai Yansheng Industrial Co, Ld). The following were the required qPCR reaction conditions: initial hold step at 95˚C for 10 min; denaturation at 95˚C for 10 s; annealing at 58˚C for 20 sec; and extension at 72˚C for 25 sec; for 40 cycles. The comparative Ct method was employed for the estimation of relative mRNA expressions of ACTN1 and ITGA5 (18). GAPDH was used as an internal parameter. All the primers were listed as follows: ACTN1, 5’-GCUGCGACAGAAGGACUAUTT-3’ (forward) and 5’-AUAGUCCUUCUGUCGCAGCTT-3’ (reverse); ITGA5 5’-CCGAGACCTGGATGGCAATGG-3’ (forward) and 5’-GGCACTAGCGGACACGATGG-3’ (reverse); GAPDH, 5’-UGACCUCAACUACAUGGUUTT-3’ (forward) and 5’-GGAGTGTTGGAGAAGTCATATTAC-3’ (reverse).

***Western blot analysis***

After indicated treatment, Tu686 cells and tumor tissues were collected and lysed in RIPA lysis buffer (Millipore). The centrifugation of lysates was implemented for the collection of supernatants. After the exposure to 10% SDS-PAGE, the transferring of proteins (20 µg/lane) to PVDF membranes (Millipore) was carried out. The overnight cultivation of membranes with primary antibodies against ACTN1, Ki-67, PCNA, MMP-2, MMP-9, E-cadherin, N-cadherin, Vimentin, ITGA5 and GAPDH was operated at 4°C, after which was the probe with proper secondary antibody (ab205718; dilution,1:2000; Abcam). The protein bands were observed with ECL reagent (Wanleibio) and the density of protein bands were quantified by the ImageJ software (v1.8; National Institutes of Health).

***Cell counting kit8 (CCK8) assay***

The inoculation of transfected Tu686 cells into 24-well plates was implemented at a density of 2×10^4^ cells/well, after which 48-hr cultivation. After that, each well was incubated with 10 μl CCK-8 reagent (CA1210, Solarbio, China). At last, the OD value was assessed in the premise of λ = 450 nm with the adoption of a microplate reader (BIO-RAD, USA) respectively at 24, 48, and 72 hr.

***EdU staining***

The transfected Tu686 cells were seeded into 6-well plates and labeled with 10 μM EdU working solution (Beyotime) for 2 hr. Subsequently, Tu686 cells were exposed to 4% paraformaldehyde fixation and 0.3% Triton X-100 permeation, followed by the cultivation with Click Additive Solution (Beyotime) for 30 min avoiding light. Hoechst 33342 was used to stain the nucleus of Tu686 cells for incubating for 10 min in the dark. At last, the proliferative ability of transfected Tu686 cells was evaluated by employing a fluorescence microscope (Olympus, Japan).

***Colony formation assay***

The inoculation of transfected Tu686 cells into 6-well plates was operated at a concentration of 1×10^5^ cells/well, after which the cultivation was done for two weeks. Following methanol fixation and Giemsa staining, the cell colonies were captured utilizing a microscope.

***Wound healing assay***

The concentration of the transfected Tu686 cells was at 1×10^5^ cells per well in the 6-well plate. When cell confluence reached 100%, a scratch was made for the transfected cells using a 200 μl pipette tip and then cultured for 24 hr. The distance between the wound edges at 0 h and 24 hr was observed by a microscope and quantified by ImageJ.

***Transwell assay***

The transfected Tu686 cells (2 × 10^4^ cells) were loaded in the upper wells coated with Matrigel (BD Biosciences, USA) of 24 well plates and a complete culture medium was added to the lower wells. After 24-hr cultivation, a cotton swab was employed to remove the cells inside the upper wells. The invaded cells were exposed to 0.1% crystal violet solution staining, then cells were coutnted adopting a light microscope (Olympus, Japan).

***Immunofluorescence***

The inoculation of transfected Tu686 cells into 24-well plates was conducted. Cells were exposed to 4% paraformaldehyde fixation as well as methanol permeation. Subsequently, the overnight cultivation of cells with a primary antibody against E-cadherin (ab40772; dilution,1:500; Abcam) was implemented at 4˚C, after which was the probe with a goat anti-rabbit IgG (HRP-conjugated) antibody (ab6802; dilution,1:1000; Abcam). A fluorescence microscope (Olympus, Japan) was adopted for the capture of cells.

***Co-immunoprecipitation (Co-IP)***

The lysis of transfected Tu686 cells was carried out on ice with RIPA lysis buffer (Shanghai Absin Biotechnology Co, Ltd). The rinse of equal amounts of protein (500 µg) with protein A/G agarose beads (Thermo Fisher Scientific, Inc) was operated, following which was the exposure to IgG (control antibody, ab172730; dilution, 1:1000; Abcam) or ACTN1 antibody (ab68194; dilution, 1:20; Abcam) or ITGA5 antibody (ab150361; dilution, 1:50; Abcam). After being washed by RIPA buffer, the immune complexes were eluted in 2X SDS-containing sample buffer at 100°C for 5 min. Western blot analysis was utilized for the estimation of ACTN1 as well as ITGA5.

***Immunohistochemistry (IHC) staining***

The tumor tissues were exposed to 4% paraformaldehyde fixation, followed by gradient ethanol dehydration and paraffin embedding. The paraffin-embedded tissues were sectioned into 5-μm thick slices, which were treated with citric acid buffer for 20 min in a water bath. Then, the 5-μm thick slices were impeded by 5% normal goat serum and then exposed to anti-ACTN1 antibody (ab155480; dilution,1:1000; Abcam), following which was the cultivation with secondary antibody (ab6721; 1:1000; Abcam). After that, the slices were rinsed with PBS, followed by DAB solution (CWBIO) incubation and then the counterstain with hematoxylin (Boster). At last, the contents of Ki-67 in tumor tissues was examined with the help of a light microscope (Olympus, Japan).

***Statistical analysis***

Data displayed as mean ± SD from at least three independent experiments were analyzed by adopting GraphPad Prism 8.0. All data were detected by Shapiro–Wilk (S–W) to evaluate whether they fit the normal distribution. If the data conformed to the normal distribution, comparisons of data between the two groups were made applying unpaired Student’s t-test and those of multiple groups were made utilizing one-way analysis of variance (ANOVA) followed by a Tukey’s *post hoc* test. *P*<0.05 meant that these experimental figures were of statistical significance.

## Results

***ACTN1 is highly expressed in HNSCC and is associated with poor prognosis***

**Figure 1:**
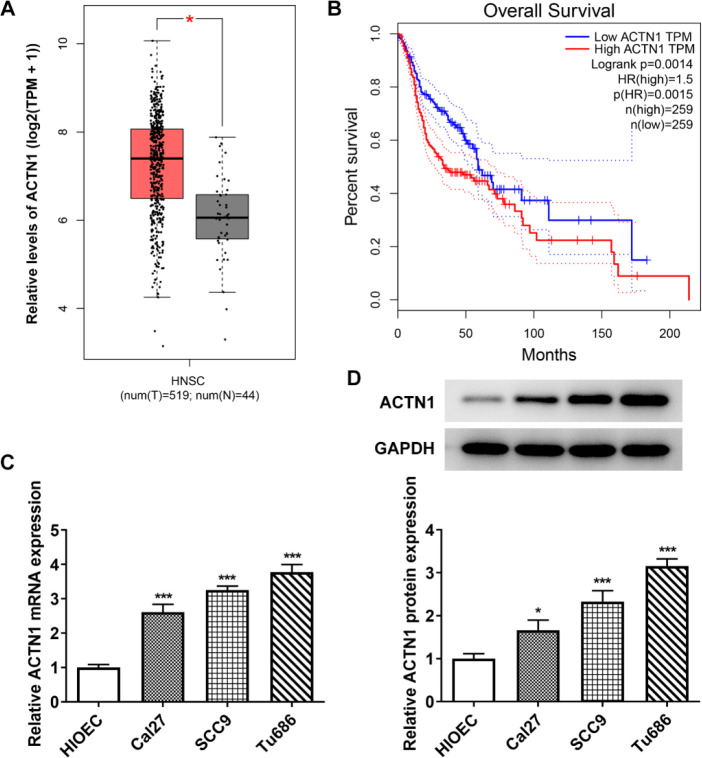
ACTN1 is highly expressed in HNSCC and is associated with poor prognosis. (A) ACTN1 expression in HNSCC tissues from GEPIA database. (B) The relation between ACTN1 expression and overall survival. (C and D) ACTN1 mRNA and protein expression in HNSCC cells transfected with shRNA-ACTN1 were detected by RTqPCR and Western blot analysis. *P<0.05 and ***P<0.001 vs. HIOEC group

ACTN1: α-Actinin 1; HNSCC: Head and neck squamous cell carcinoma; HIOEC: Human immortalized oral epithelial cell; GEPIA: Gene expression profiling interactive analysis

GEPIA database demonstrated that ACTN1 expression was conspicuously ascended in HNSCC in comparison with the normal control (Figure 1A). ACTN1 upregulation had a significant association with the overall survival of patients with HNSCC (Figure 1B). The ACTN1 expression was increased in HNSCC cells than in HIOEC and the highest in Tu686 cells (Figures 1C and 1D). In this way, the Tu686 cell line was adopted for the ensuing study.

***Knockdown of ACTN1 inhibits the proliferation of HNSCC cells***

**Figure 2:**
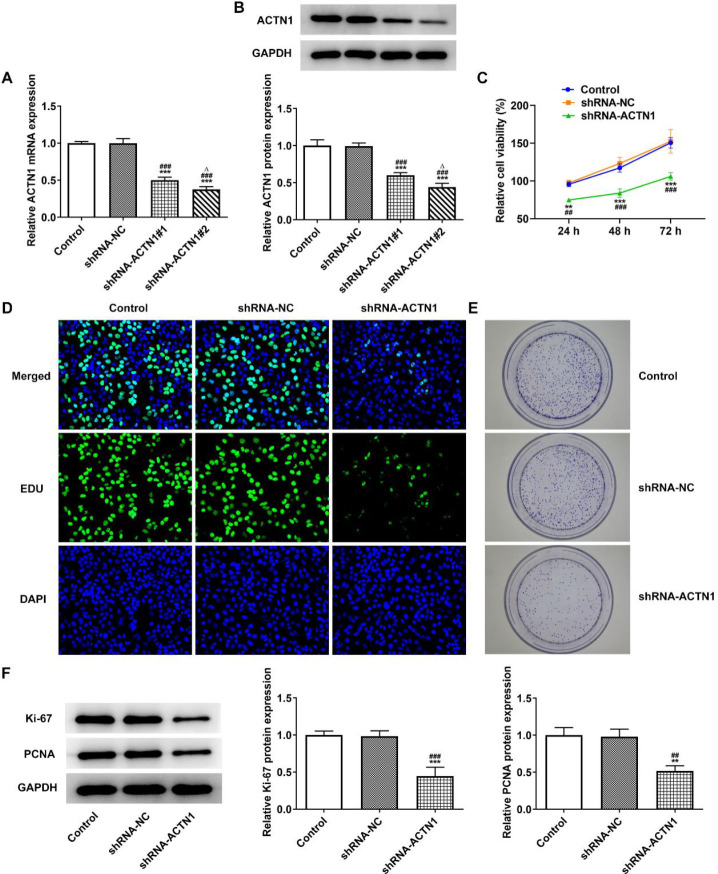
Knockdown of ACTN1 inhibits proliferation of HNSCC cells. (A and B) ACTN1 mRNA and protein expression in Tu686 cells transfected with shRNA-ACTN1 were detected by RTqPCR and Western blot analysis. ***P<0.001 vs. Control group. ###*P*<0.001 vs. shRNA-NC group. ∆*P*<0.05 vs. shRNA-ACTN1#1 group. The viability (C), proliferation (D) and colony formation ability (E) of Tu686 cells transfected with shRNA-ACTN1 were detected by CCK-8 assay, EdU staining and colony formation assay respectively. (F) The expression of proliferation related proteins in Tu686 cells transfected with shRNA-ACTN1 was detected by Western blot analysis. **P<0.01 and ***P<0.001 vs. Control group. ##*P*<0.01 and ###*P*<0.001 vs. shRNA-NC group

ACTN1: α-Actinin 1; HNSCC: Head and neck squamous cell carcinoma

After the transfection with shRNA-ACTN1#1/2, ACTN1 expression in Tu686 cells was descended. The ACTN1 expression was lower in the shRNA-ACTN1#2 group than in the shRNA-ACTN1#1 group, thereby choosing the shRNA-ACTN1#2 for the next experiment (Figures 2A and 2B). Knockdown of ACTN1 suppressed the viability (Figure 2C), proliferation (Figure 2D) as well as colony forming ability (Figure 2E) of Tu686 cells. Correspondingly, the expressions of Ki-67 and PCNA in Tu686 cells were declined by knockdown of ACTN1 (Figure 2F).

***Knockdown of ACTN1 inhibits invasion, migration and EMT of HNSCC cells***

**Figure 3:**
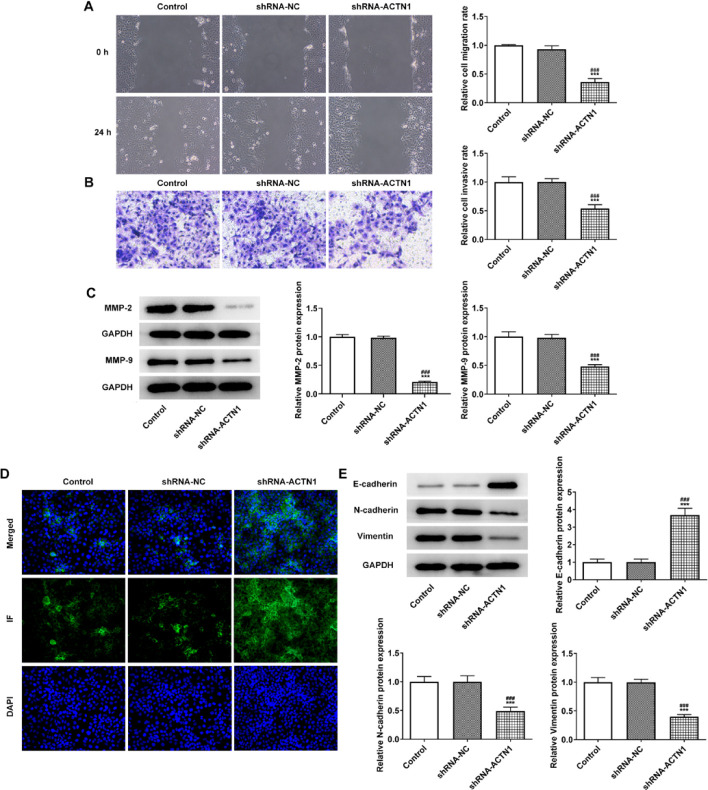
Knockdown of ACTN1 inhibits invasion, migration and EMT of HNSCC cells

ACTN1 depletion suppressed the migrative and invasive capabilities of Tu686 cells (Figures 3A and 3B) and descended the contents of MMP-2 and MMP-9 (Figure 3C). The result of immunofluorescence indicated that E-cadherin expression was enhanced in shRNA-ACTN1 transfected Tu686 cells (Figure 3D) and western blot analysis also showed that knockdown of ACTN1 promoted the E-cadherin expression while inhibited the expression of N-cadherin and Vimentin in Tu686 cells (Figure 3E).

***ITGA5 is highly expressed in HNSCC and interacts with ACTN1***

**Figure 4:**
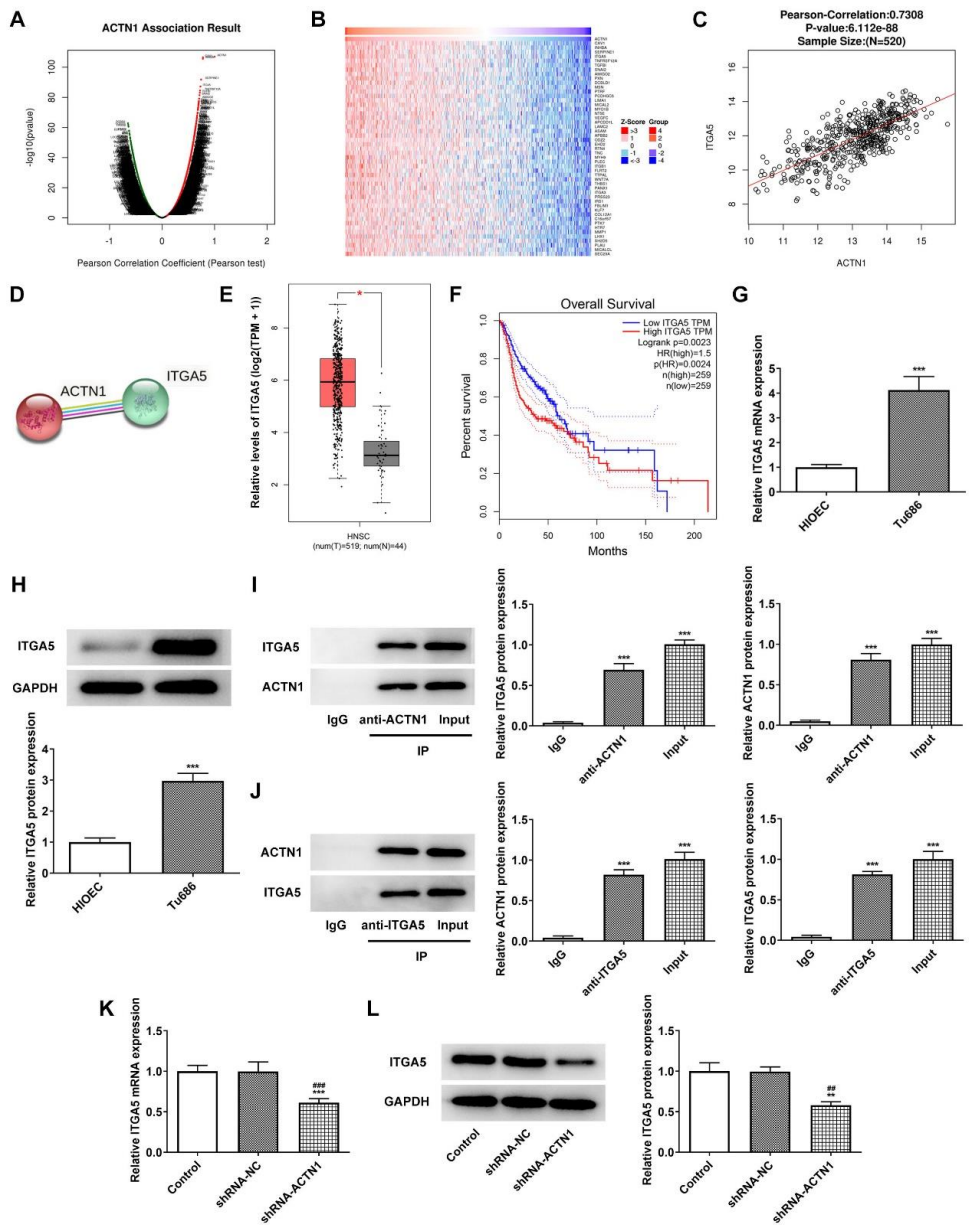
Overexpression of ITGA5 reverses the inhibitory effect of ACTN1 knockdown on the proliferation of HNSCC cells. (A-C) ACTN1 was positively correlated with ITGA5 in the LinkedOmics database. (D) STRING database showed a potential interaction between ACTN1 and ITGA5. (E) ITGA5 expression in HNSCC tissues from GEPIA database. (F) The relation between ITGA5 expression and overall survival. (G and H) ITGA5 mRNA and protein expression in Tu686 cells were detected by RTqPCR and Western blot analysis. ***P<0.001 vs. HIOEC group. (I and J) The interaction between ACTN1 and ITGA5 was confirmed by co-immunoprecipitation. ***P<0.001 vs. IgG group. (K and L) ITGA5 mRNA and protein expression in Tu686 cells transfected with shRNA-ACTN1 were detected by RTqPCR and Western blot analysis. **P<0.01 and ***P<0.001 vs. Control group. ##*P*<0.01 and ###*P*<0.001 vs. shRNA-NC group

ITGA5: Integrin α5; ACTN1: α-Actinin 1; HNSCC: Head and neck squamous cell carcinoma

LinkedOmics database showed that ACTN1 was positively associated with ITGA5 (Figure 4A-C). The STRING database also found a potential interaction between ACTN1 and ITGA5 (Figure 4D). GEPIA database demonstrated that ITGA5 expression was greatly enhanced in HNSCC when compared to the normal control (Figure 4E). ITGA5 upregulation was also markedly related to the overall survival of patients suffering from HNSCC (Figure 4F). Results in Figure 4G and 4H presented that the expression of ITGA5 was upregulated in Tu686 cells compared with HIOEC. After the addition of anti-ACTN1 and anti-ITGA5 into the cell lysate, the expression of ACTN1 and ITGA5 was all seen, which indicated that ACTN1 could interact with ITGA5 (Figure 4I and 4J). After transfection with shRNA-ACTN1, ITGA5 expression in Tu686 cells was decreased (Figures 4K and 4L).

***Overexpression of ITGA5 reverses the inhibitory effect of ACTN1 knockdown on the proliferation of HNSCC cells***

**Figure 5:**
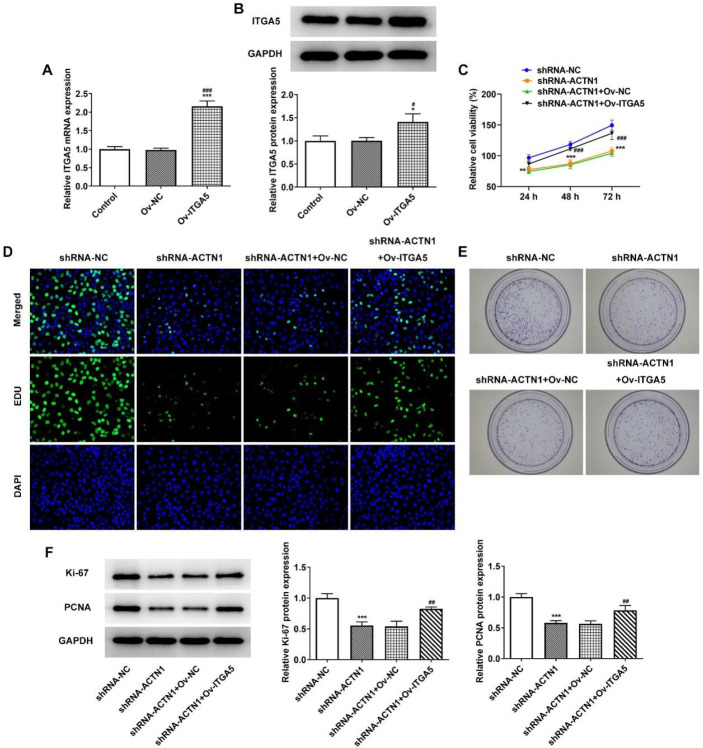
Overexpression of ITGA5 reverses the inhibitory effect of ACTN1 knockdown on the proliferation of HNSCC cells. (A and B) ITGA5 mRNA and protein expression in Tu686 cells transfected with ov-ITGA5 were detected by RTqPCR and Western blot analysis. *P<0.05 and ***P<0.001 vs. Control group. #*P*<0.05 and ###*P*<0.001 vs. shRNA-NC group. ∆*P*<0.05 vs. shRNA-ACTN1#1 group. The viability (C), proliferation (D) and colony formation ability (E) of Tu686 cells transfected with shRNA-ACTN1 and ov-ITGA5 were detected by CCK-8 assay, EdU staining and colony formation assay, respectively. (F) The expression of proliferation-related proteins in Tu686 cells transfected with shRNA-ACTN1 and ov-ITGA5 was detected by Western blot analysis. **P<0.01 and ***P<0.001 vs. shRNA-NC group. ##*P*<0.01 and ###*P*<0.001 vs. shRNA-ACTN1+ov-NC group

ITGA5: Integrin α5; ACTN1: α-Actinin 1; HNSCC: Head and neck squamous cell carcinoma

After transfection with Ov-ITGA5, ITGA5 expression in Tu686 cells was elevated (Figures 5A and 5B). ITGA5 overexpression improved the viability, proliferation and colony formation of shRNA-ACTN1 transfected Tu686 cells (Figures 5C-E). Also, the expression of Ki-67 and PCNA in shRNA-ACTN1 transfected Tu686 cells was upregulated by ITGA5 overexpression (Figure 5F).

***Overexpression of ITGA5 reverses the inhibitory effect of ACTN1 knockdown on invasion, migration as well as EMT of HNSCC cells***

**Figure 6:**
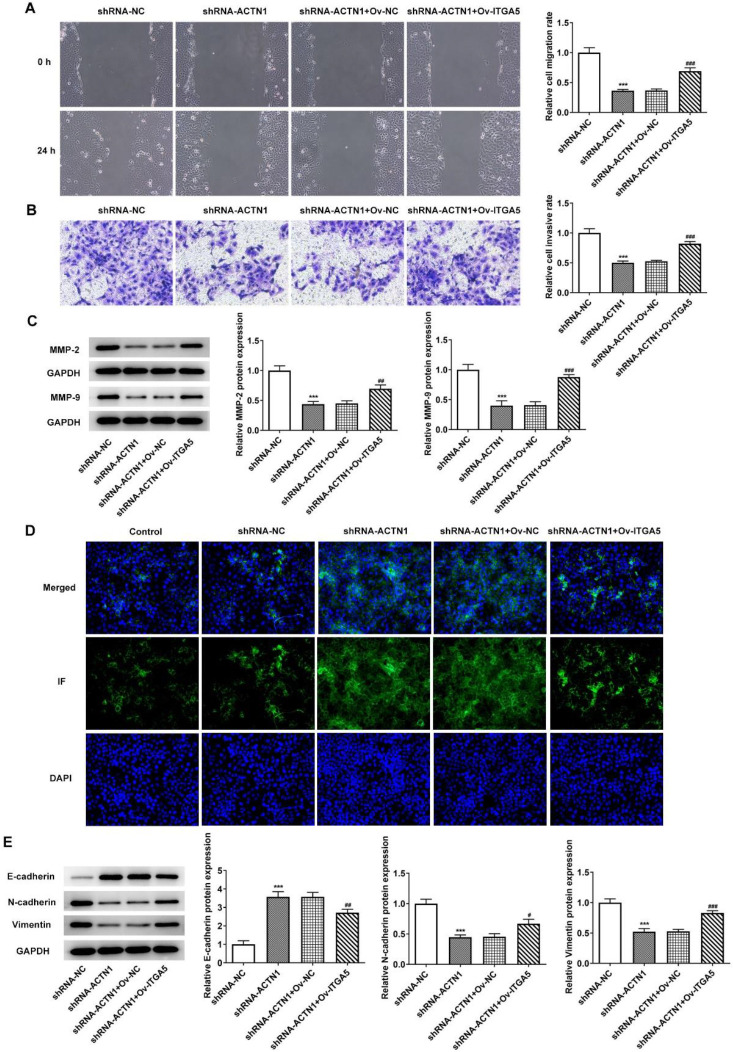
Overexpression of ITGA5 reverses the inhibitory effect of ACTN1 knockdown on invasion, migration and EMT of HNSCC cells. The migration (A) and invasion (B) of Tu686 cells transfected with shRNA-ACTN1 and ov-ITGA5 was detected by wound healing assay and transwell assay. (C) The expression of metastasis-related proteins in Tu686 cells transfected with shRNA-ACTN1 and ov-ITGA5 was detected by Western blot analysis. (D and E) The expression of EMT-related proteins in Tu686 cells transfected with shRNA-ACTN1 and ov-ITGA5 was detected by immunofluorescence and western blot analysis. ***P<0.001 vs. shRNA-NC group. #*P*<0.05, ##*P*<0.01 and ###*P*<0.001 vs. shRNA-ACTN1+ov-NC group

ITGA5: Integrin α5; ACTN1: α-Actinin 1; HNSCC: Head and neck squamous cell carcinoma; EMT: Epithelial-mesenchymal transformation

ITGA5 overexpression also enhanced the migration and invasion of shRNA-ACTN1 transfected Tu686 cells (Figures 6A and 6B) and promoted the contents of MMP-2 as well as MMP-9 (Figure 6C). The E-cadherin expression decreased in Tu686 cells co-transfected with shRNA-ACTN1 and Ov-ITGA5 than that of single shRNA-ACTN1 (Figure 6D). As Figure 6E depicted, ITGA5 overexpression downregulated the E-cadherin expression and upregulated the expression of N-cadherin and Vimentin in shRNA-ACTN1 transfected Tu686 cells.

***Knockdown of ACTN1 inhibits growth and metastasis of HNSCC in vivo***

**Figure 7:**
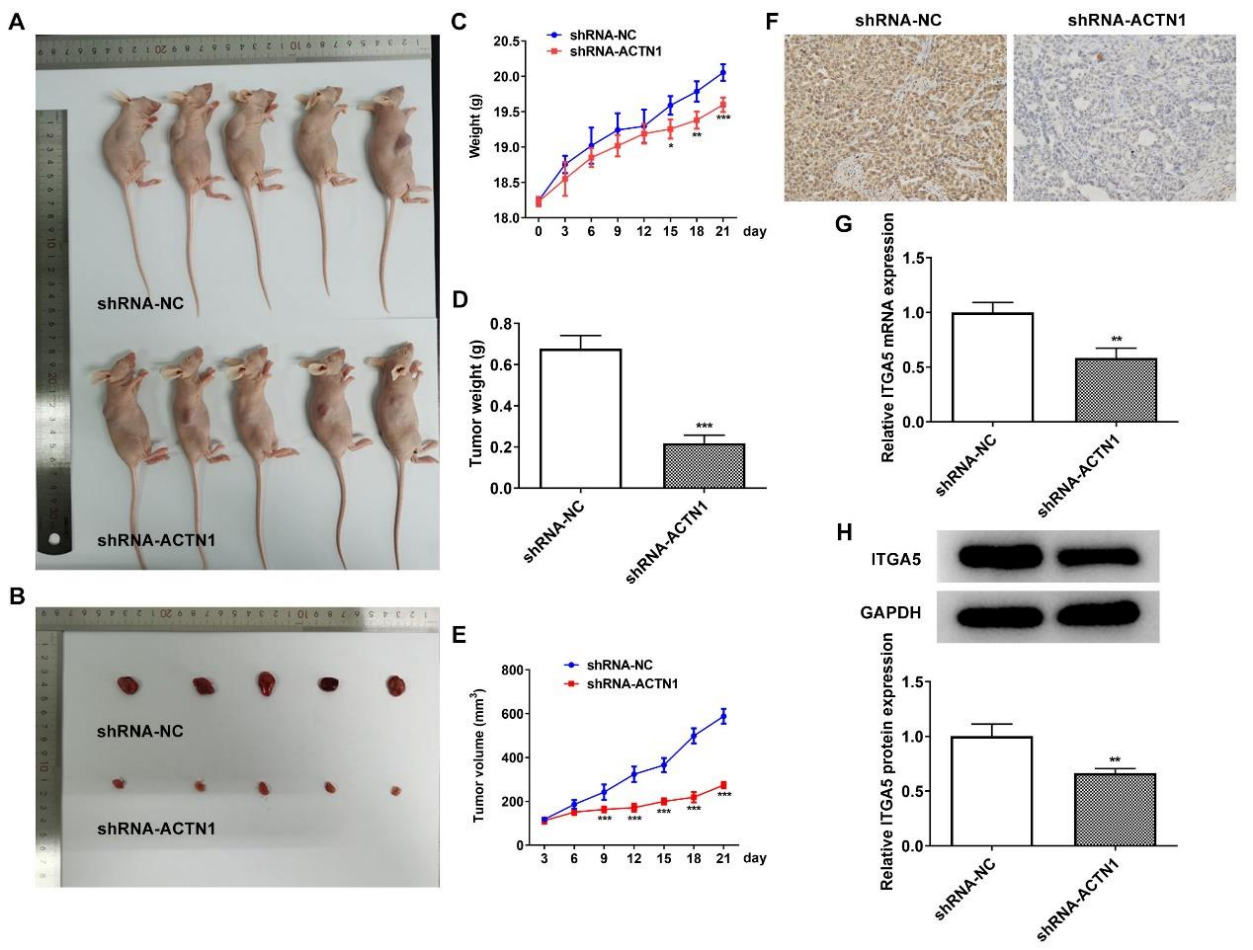
Knockdown of ACTN1 inhibits growth and metastasis of HNSCC in vivo. (A and B) The appearances of mice and tumor in the two groups. (C) Mice weight changed from day 0 to day 21. (D) Tumor weight in mice transfected with shRNA-ACTN1 was determined. (E) Tumor volume changed from day 0 to day 21. (F) The expression of Ki-67 in tumor tissues was analyzed by immunohistochemistry staining. (G and H) ITGA5 mRNA and protein expression in mice transfected with shRNA-ACTN1 were detected by RTqPCR and Western blot analysis. *P<0.05, **P<0.01 and ***P<0.001 vs. shRNA-NC group

ACTN1: α-Actinin 1; HNSCC: Head and neck squamous cell carcinoma

The morphology of mice and tumor tissues were shown as Figures 7A and 7B. The weight of mice transfected with shRNA-ACTN1 was different from that transfected with shRNA-NC from Day 15 to 21 (Figure 7C). After transfection with shRNA-ACTN1, the weight and volume of tumor in mice were all diminished (Figures 7D and 7E). The contents of Ki-67 (Figure 7F) and ITGA5 (Figures 7G and 7H) in tumor tissues were downregulated in mice transfected with shRNA-ACTN1.

The migration (A) and invasion (B) of Tu686 cells transfected with shRNA-ACTN1 were detected by wound healing assay and transwell assay. (C) The expression of metastasis-related proteins in Tu686 cells transfected with shRNA-ACTN1 was detected by Western blot analysis. (D and E) The expression of EMT-related proteins in Tu686 cells transfected with shRNA-ACTN1 was detected by immunofluorescence and western blot analysis. ***P<0.001 vs. Control group. ###*P*<0.001 vs. shRNA-NC group

ACTN1: α-Actinin 1; EMT: Epithelial-mesenchymal transformation; HNSCC: Head and neck squamous cell carcinoma

## Discussion

At present, the effective therapies for HNSCC include surgical resection, radiotherapy, chemotherapy, targeted therapy and immunotherapy. Despite the fact that certain progress has been achieved in these aspects, the overall 5-year survival rate has not been greatly enhanced (19). Most patients are often diagnosed with advanced HNSCC and have high recurrence rates and poor prognoses (20). Therefore, exploring effective diagnostic and prognostic biomarkers is the key to improving the therapeutic efficacy, long-term survival rate and life quality of patients, and acts as a vital player in driving the advancement of HNSCC treatment.

The α-Actinin protein plays a critical role in the ability of cells to move and migrate (21, 22). ACTN4 is often associated with the occurrence and development of various tissue types of tumors, such as colon cancer, breast cancer, pancreatic cancer and ovarian cancer, which proved that the high expression of ACTN4 is closely related to the metastatic and invasive ability of tumors (23-27). Previous studies also indicated that ACTN1 knockdown could inhibit the metastasis of breast cancer and oral squamous cell carcinoma (11, 12). Interference of ACTN1 could improve the survival rate of xenograft tumor rats (9). Here, we speculated that ACTN1 might also be functioning in HNSCC. We have confirmed that the expression of ACTN1 in tumor tissues of HNSCC patients was increased, which had a relation with the low overall survival. In addition, ACTN1 expression was also high in HNSCC cells and downregulation of ACTN1 could also suppress cell proliferative, migrative and invasive abilities as well as EMT in HNSCC, and reduce the tumor growth in xenograft tumor mice, which was in line with the role of ACTN1 in other tumors.

As a transmembrane protein, integrins regulate cell proliferation and migration by acting as a link between different cells or the communication between cells and the extracellular matrix (28). It has been confirmed that integrin is involved in the growth, invasion and metastasis of various malignancies (29). ITGA5 acted as a regulatory player in biological functions, including cell adhesion, proliferation, apoptosis and motility, by binding to extracellular matrix proteins through the extracellular structure (30, 31). Studies have confirmed that ITGA5 is highly expressed in gastric, breast, and ovarian cancer, which can be used as a prognostic tumor marker and is closely related to the adverse prognosis of patients (32-34). ITGA5 had abundant existence in gastric cancer, and its upregulation correlated with the survival prognosis of patients (32). Human mesenchymal stem cells could promote migration and invasion of HCC cells by targeting ITGA5 regulation (35). ITGA5 could promote the adhesion of cancer cells to the bone in breast cancer, which is related to bone metastasis of breast cancer (36). In gastric cancer, target suppression of ITGA5 resulted in a great reduction in the invasive and migrative abilities of gastric cancer cells (37). ITGA5 promoted cell proliferative, migrative and invasive abilities in oral squamous cell carcinoma (OSCC) by regulating PI3K/AKT signaling pathway, thus promoting the malignant advancement of o OSCC (38). In this study, ITGA5 expression was also increased in tumor tissues of HNSCC patients, which had a relation with low overall survival. Furthermore, ITGA5 overexpression could also promote cell proliferative, migrative and invasive abilities and EMT in HNSCC, weakening the effects of ACTN1 knockdown.

Arf6 guanine-nucleotide exchange factor (CYTH2) interacts with the ACTN1 to regulate cellular Arf6 activity involved in neurite extension (39). PRDM1A acts upstream of ITGA5 to regulate the posterior pharyngeal arch development in zebrafish (40). miR-27b/ ITGA5 axis participated in the regulation of tongue squamous cell carcinoma epithelial-mesenchymal transition (16) and lncRNA NEAT1/ miR-128-3p/ITGA5 axis was involved in the regulation of glioma progression (41). CHI3L1/ITGA5 axis was related to treating atopic dermatitis (42). GBX2 binding to the ITGA5 promoter promotes the viability, migration, and invasion of bladder cancer cells (43). We found that ACTN1 and ITGA5 could interact with other genes to regulate some diseases and this study first explored the interaction between ACTN1 and ITGA5 in the regulation of cancer progression. The LinkedOmics and STRING databases were used to predict the interaction between ACTN1 and ITGA5 in HNSCC cells, and a Co-IP experiment was conducted to confirm the interaction.

## Conclusion

It was found that the ACTN1 and ITGA5 were highly expressed in HNSCC tissues and cells. ACTN1 knockdown inhibited cell proliferative, migrative and invasive abilities as well as EMT in HNSCC, which were reversed by ITGA5 overexpression. What’s more, ACTN1 depletion also repressed the growth and metastasis of tumor tissues in tumor xenografts of nude mice. The present study may provide a potential biomarker for diagnosing and treating HNSCC.

## Authors’ Contributions

HZ and RW designed the study, and RW drafted and HZ revised the manuscript. RW and YG performed the experiments, analyzed the data and searched the literature. All authors read and approved the final manuscript.

## Funding

None.

## Conflicts of Interest

The authors declare they have no competing interests.
